# Preparation by mandatory E-modules improves learning of practical skills: a quasi-experimental comparison of skill examination results

**DOI:** 10.1186/s12909-015-0376-4

**Published:** 2015-06-10

**Authors:** Kelly J. Kwant, Eugene J. F. M. Custers, Femke J. Jongen-Hermus, Manon Kluijtmans

**Affiliations:** 1 Department Clinical Skills Training, University Medical Center Utrecht, HB Building 3.06, P.O. Box 85500,, 3508 Utrecht, GA The Netherlands; 2 Center for Research and Development of Education, University Medical Center Utrecht, HB Building 4.05, P.O. Box 85500,, 3508 Utrecht, GA The Netherlands

**Keywords:** E-learning, Blended learning, Skills education, Undergraduate education

## Abstract

**Background:**

Until recently, students at UMC Utrecht Faculty of Medicine prepared for practical skills training sessions by studying recommended literature and making written assignments, which was considered unsatisfactory. Therefore, mandatory e-modules were gradually introduced as substitute for the text based preparation. This study aimed to investigate whether this innovation improved students’ performance on the practical skills (OSCE) examination.

**Method:**

In both the 2012 and 2013 OSCEs, e-modules were available for some skill stations whereas others still had text based preparation. We compared students’ performance, both within and between cohorts, for skill stations which had e-module preparation versus skill stations with text based preparation.

**Results:**

We found that performance on skill stations for which students had prepared by e-modules was significantly higher than on stations with text based preparation, both within and between cohorts. This improvement cannot be explained by overall differences between the two cohorts.

**Conclusion:**

Our results show that results of skills training can be improved, by the introduction of e-modules without increasing teacher time. Further research is needed to answer the question whether the improved performance is due to the content of the e-modules of to their obligatory character.

## Background

Before medical students start their first clerkships, they have to be thoroughly trained in physical examination skills. Most medical programmes have introduced practical skills training sessions. Skills training requires practice materials, rooms, and a high teacher-student ratio, which makes skill training very resource intensive. It is important to use time and recourses available for skills training as efficiently as possible, to optimize learning outcomes.

The medical curriculum of UMC Utrecht Faculty of Medicine is a six year programme characterized by early patient-contact: the medical students in Utrecht start with their first full-fledged clerkship in the third year. To prepare the students for these early clerkships, in the first two years of the programme students learn how to perform the basics of physical examination (e.g., examination of lungs, elbow or neurological examination) and other basic medical skills (e.g., intramuscular injections or resuscitation). In addition to these supervised training sessions students have the possibility to practice and further improve their skills by unsupervised practice in a skills lab, as well as the possibility to consult the skills teachers with any questions they have by using the electronic learning environment and by appointment. Even with these additional training possibilities, in all likelihood the regular training sessions are the most important element in mastering the skills, because it is the only preparation that involves modeling of a skill by a trainer.

Students are required to prepare for the training sessions, in order to keep time for instructions to a minimum, leaving as much time as possible to actually practice the skills under supervision. Until recently, students had to prepare by studying recommended literature and make assignments about the theoretical, practical and clinical background of the skill. In the rest of the paper we will refer to this type of preparation as ‘text based preparation’. Due to time limitations these written assignments were not discussed in detail in the training sessions and students did not receive any feedback on their preparation. Thus, many students lacked motivation to thoroughly prepare (confirmed by course evaluations in which students indicate they did not prepare well). As a consequence, teachers noticed that most students arrived at the training sessions unprepared and valuable practice time was lost introducing the clinical background, the necessary anatomical knowledge and performance of the skill to the students.

In addition to the lack of student motivation, the text based preparation might not have been the best means to prepare students for a practical skills training session due to its inherently passive nature (it is impossible to exercise the skill). Therefore, in 2010 the faculty decided to gradually substitute e-modules for text based preparation. These e-modules cover the same content as the text based preparation: information about the theoretical background of the skill and its clinical application, as well as instructions on how the skill is performed in practice. However, in the e-modules this content is delivered in a richer format, including video, sound, animations and exercises with direct feedback. An e-module finishes with a formative self-test and takes students approximately 1–1.5 h to complete. The e-modules were developed specifically for our practical skills training programme. Because the development of e-modules is time and labor intensive, e-modules were developed successively over a prolonged period of time for all 16 physical examination sessions from 2011 to 2014. Thus, from academic years 2011–2012 until 2013–2014 for some skills training sessions e-modules were available, whereas for other skills training sessions, students still used text based preparation.

Besides being more appealing to students, the digital nature of e-modules allows teachers to check whether the students have completed the e-module. At our faculty, completion of the modules is mandatory before students are allowed to participate in the training session. In contrast, for the text based preparation it was practically not possible to check whether students had prepared for their skill training session. Although completing the e-modules is obligatory, and completion is checked in the electronic learning environment before each training session, no control is exerted as to how thoroughly the students study the issues addressed. Students have to proceed through the e-module linearly and can’t skip topics or interactive elements the first time. After completion of the e-module (which is registered in the electronic learning environment), the e-module remains available to the student.

The addition of e-modules to a practical training session is a form of blended learning. Blended learning has consistently been shown to have a high learning satisfaction and either an equal or more positive effect on theoretical knowledge acquisition and clinical reasoning [1–5]. For practical skills training preparation, however, few studies have been published. Bloomfield and Jones [6] compared student satisfaction in learning clinical skills with a blended learning approach versus a traditional approach. In this research, students did think e-modules could be valuable for developing clinical skills, but they did not want to relinquish practical training sessions. Two other studies, by Arroyo-Morales et al. [7] and Orientale et al. [8], showed that the availability of media material, like videos, in addition to the face-to-face lessons, could improve the performance on skills, like palpation of the knee or other physical examination skills. However, their e-learning did not include interactive elements.

This study aimed to investigate whether obligatory interactive e-modules as preparation for practical skills training sessions led to better performance of the skills by students than training sessions with text based preparation. The learning outcome was measured by the average scores obtained at a 2-station Observed Structured Clinical Exam (OSCE) for physical examination skills at the end of each academic year. This is a high-stakes examination where all students will try to perform to the best of their abilities, because admittance to their clinical rotations is conditional on passing this OCSE. In the OSCE we measure the final level at which students have mastered these skills. This level depends not solely on preparation and training sessions, because in addition to re-using the e-module, students have other opportunities to practice these skills to prepare themselves for the OSCE, e.g. exercising the skills on fellow students [9,10]. The time lag between the practical training sessions and the OSCE ranged from a few weeks up to 20 months. This latter time lag would occur if a student attended the practical training of a particular skill in the beginning of year 1 and was tested for this skill in the OSCE at the end of year 2. Given this time lag and the array of opportunities students had to prepare themselves for the OSCE, it could be expected that we were only able to find small differences between students’ performance on skills they prepared text based versus e-modules. However, if we would find higher OSCE scores for students tested on skills for which e-modules were available than for skills with text based preparation, this would be evidence that use of e-modules could result in better performance of the skills tested by the OSCE. In other words, we predict that the OSCE exam has a higher overall score in 2012–2013 due to the introduction of more e-modules in that year.

## Method

Our study covered the academic years 2011–2012 and 2012–2013. In both years for part of the skill training sessions e-modules were available whereas for other sessions students still used text based preparation. In years 1 and 2, a total of 27 training sessions were scheduled: 16 physical examination training sessions, and 11 other basic medical skills training sessions (see Appendix). Each training session focused on a specific skill or set of skills and started with a an instruction and a demonstration by a teacher to groups of eight students. Subsequently, the students practiced the skill in pairs, either on each other or using training materials (medical phantoms and low fidelity simulations e.g. intravenous practice arms). The duration of each training session was 1 h and 45 min. All e-modules dealt with physical examination skills. As a result, our study was limited to these skills.

Participants were all first and second year medical students at the UMC Utrecht who participated in the end of year 2-station OSCE. In this OSCE two different skills were tested in a 12 min time frame. The intervention was part of the regular quality improvement cycle, and the collected data were part of the routine educational assessment. Therefore, approval from the ethical committee of the Dutch Society of Medical Education was not necessary.

Data (OSCE scores, see Table 1) were collected from 15 stations in year 1, and 15 stations in year 2, so 30 stations in total. Our main unit of analysis was the individual student score. The individual student score of each station consisted of an average of 3 to 6 sub-items - depending on the station-, each sub-item being scored on a 5-point scale. Theoretically the individual student scores could range from 1.00 to 5.00. The total number of students in the dataset was 780, each participating in 2 to 4 stations. In total we were able to collect data from 2010 individual student scores from which we calculated the average scores per station (see Tables 2 and 3).

**Table 1:** Details of the 2-station OSCE in year 1 and 2

Students take a 2-station OSCE for physical examination skills twice during the first 2 years of the medical curriculum, at the end of year 1 and 2^a^. In each examination two skills are tested, chosen from a pool of 18 skills for year 1 and 21 skills for year 2. The stations are assigned non-systematically. Students performance on each station is observed by a trained and experienced examiner, and assessed by using several sub-items scored on a 5-point scale. From these sub-items an average score was calculated.		
5 point scale used during 2-station OSCE		
1^b^	Poor	Error which is dangerous for a patient, e.g. feeling the carotid artery both sides at the same time.
2	Insufficient	Bad execution of the sub-item or sub-item is not shown
3	Moderate	Showed the sub-item, but with remarks on execution
4	Sufficient	Only small remarks on execution
5	Good	No remarks, perfect execution

^a^At the end of year 2 the basic medical skills are tested in a 1- station OSCE

^b^Only given in exceptional situations. During this study it did not occur

**Table 2:** Scores for stations at 2013 examination year 1 and 2 (different stations, within cohort)

Station		Written preparation	Station		E-module preparation
*Year 1*	*General examination*	*3.79* ± *0.57 (n = 69)*	*Year 1*	*Head and neck 2*	*3.88* ± *0.61 (n = 31)*
	*Neurology arms: inspection*	*3.36* ± *0.81 (n = 34)*		*Spine*	*3.53* ± *0.68 (n = 31)*
	*Neurology legs: reflexes*	*3.72* ± *0.61 (n = 35)*		*Lungs 1*	*3.48* ± *0.77 (n = 44)*
	*Cranial nerves*	*3.39* ± *0.74 (n = 44)*		*Lungs 2*	*3.59* ± *0.82 (n = 33)*
	*Neurology arms: reflexes*	*3.74* ± *0.52 (n = 31)*		*Elbow*	*3.72* ± *0.90 (n = 33)*
	*Neurology legs: inspection*	*3.65* ± *0.63 (n = 34)*		*Heart*	*3.63* ± *0.66 (n = 31)*
	*Neurology: gait*	*3.67* ± *0.73 (n = 35)*		*Abdomen 1*	*3.54* ± *0.76 (n = 34)*
				*Abdomen 2*	*3.50* ± *0.59 (n = 35)*
Year 2	General examination	3.89 ± 0.50 (n = 72)	Year 2	Knee	3.52 ± 0.68 (n = 29)
	Cranial nerves	3.49 ± 0.69 (n = 40)		Thorax 1	3.75 ± 0.62 (n = 37)
	Neurology: gait	3.16 ± 0.69 (n = 32)		Thorax 2	3.64 ± 0.80 (n = 30)
	Peripheral circulation	3.14 ± 0.60 (n = 37)		Neurology arms: inspection	3.85 ± 0.72 (n = 30)
	Spine	3.26 ± 0.65 (n = 31)		Neurology arms: reflexes	3.83 ± 0.57 (n = 24)
	Head and neck 1	3.70 ± 0.66 (n = 29)		Neurology legs: inspection	3.82 ± 0.60 (n = 28)
	Head and neck 2	3.62 ± 0.56 (n = 31)		Abdomen 1	3.83 ± 0.72 (n = 24)
				Abdomen 2	3.72 ± 0.61 (n = 28)
	Mean (SD)	3.58 ± 0.67 (n = 554)		Mean (SD)	3.67 ± 0.71 (n = 502)
				p-value	0.03

*Italics: Year 1*

Normal: Year 2

**Table 3:** Scores for stations in 2012 and 2013 examination year 1 and 2 (same stations, between cohorts)

		Intervention group (E-module introduction 2013)				Control group	
	Station	2012 Written preparation N = 308	2013 E-module preparation N = 314		Station	2012 Written preparation N = 467	2013 Written preparation N = 517
*Year1*	*Head and neck 2*	*3.44 ± 0.60*	*3.88 ± 0.61*	*Year 1*	*General examination*	*3.81 ± 0.62*	*3.79 ± 0.57*
		*(n = 35)*	*(n = 31)*			*(n = 65)*	*(n = 69)*
	*Elbow*	*3.63 ± 0.58*	*3.72 ± 0.90*		*Neurology arms: inspection*	*3.42 ± 0.78*	*3.36 ± 0.81*
		*(n = 33)*	*(n = 33)*			*(n = 34)*	*(n = 34)*
	*Heart*	*3.47 ± 0.81*	*3.63 ± 0.66*		*Neurology legs: reflex*	*3.13 ± 0.55*	*3.72 ± 0.61*
		*(n = 35)*	*(n = 31)*			*(n = 31)*	*(n = 35)*
	*Abdomen 1*	*3.41 ± 0.65*	*3.54 ± 0.76*		*Cranial nerves*	*3.48 ± 0.63*	*3.39 ± 0.74*
		*(n = 33)*	*(n = 34)*			*(n = 32)*	*(n = 44)*
	*Abdomen 2*	*3.34 ± 0.62*	*3.50 ± 0.59*		*Neurology arms: reflex*	*3.61 ± 0.64*	*3.74 ± 0.52*
		*(n = 33)*	*(n = 35)*			*(n = 35)*	*(n = 31)*
					*Neurology legs: inspection*	*3.60 ± 0.70*	*3.65 ± 0.63*
						*(n = 33)*	*(n = 34)*
					*Neurology: gait*	*3.64 ± 0.80*	*3.67 ± 0.73*
						*(n = 33)*	*(n = 35)*
Year 2	Knee	3.12 ± 0.55	3.52 *±* 0.68	Year 2	General examination	3.82 *± 0.46*	3.89 *±* 0.50
		(n = 28)	(n = 29)			*(n = 48)*	(n = 72)
	Thorax 1	3.77 ± 0.45	3.75 *±* 0.62		Cranial nerves	3.65 *± 0.72*	3.49 *±* 0.69
		(n = 29)	(n = 37)			*(n = 19)*	(n = 40)
	Thorax 2	3.72 ± 0.58	3.64 *±* 0.80		Neurology: Gait	3.47 *± 0.72*	3.16 *±* 0.69
		(n = 27)	(n = 30)			*(n = 49)*	(n = 32)
	Neurology arms: inspection	3.40 ± 0.60	3.85 *±* 0.72		Spine	3.04 *± 0.46*	3.26 *±* 0.65
		(n = 27)	(n = 30)			*(n = 30)*	(n = 31)
	Neurology arms: reflex	3.05 ± 0.49	3.83 *±* 0.57		Head and neck 1	3.69 *± 0.61*	3.70 *±* 0.66
		(n = 28)	(n = 24)			*(n = 28)*	(n = 29)
					Head and neck 2	3.72 *± 0.57*	3.62 *±* 0.56
						*(n = 30)*	(n = 31)
	Mean (SD)	3.44 ± 0.63	3.68 ± 0.70		Mean (SD)	3.57 ± 0.67	3.61 ± 0.67
	p-value	<0.01			p-value	0.34	

*Italics: Year 1*

Normal: Year 2

In 2011–2012, 4 of the 30 OSCE stations had e-module preparation, whereas in 2012–2013 this had increased to 16 of the 30 OSCE stations. For a first global impression, the average score for all stations of the 2011–2012 OSCE, and for all stations of 2012–2013 OSCE were calculated and compared for difference.

For a more detailed analysis of the effect of e-module preparation, scores for stations which had e-module preparation were compared with the scores for stations which had text based preparation. Three main comparisons were made:

1. A within cohort comparison of scores for all skill stations with e-module preparation versus all stations with text based preparation (different set of stations). We expected higher average performance at stations with e-module preparation for the training session.
2. A between cohort comparison of scores before and after introduction of mandatory e-module preparation (same set of stations). To check whether a possible difference could have been caused by a cohort difference in students’ performance, we also compared average scores of stations with text based preparation for the training sessions in both years. Because in 2011–2012 only four stations had e-module preparation, we had few data to compare the results of stations that had e-module preparation in both 2011–2012 and 2012–2013. We predicted higher scores for stations which changed from text based preparation to e-module preparation, but no difference on scores for stations for which students had text based preparation for both years.
3. A within participants comparison (different stations)*.* Because at our OSCE, students were non-systematically assigned to the stations, it could occur that a student was examined on: two stations which both had text based preparation, two stations which both had e-module preparation, or one station with text based preparation and one station with e-module preparation. For the last group of students (332 students) we compared the individual station scores between e-module versus text based preparation. We predicted that these students would have a higher score for the station with an e-module preparation.

### Data-analysis

The distribution of station scores was tested for normality. As this distribution did depart significantly from normality, for the text based preparation group (W = 0.988, df = 554, p < 0.0001), as well as for the e-module preparation group (W = 0.980, df = 502, p < 0.0001); the appropriate test would be a non-parametric test. However, as the data covered a wide range of decimal values on the 5-point scale and the distribution was unimodal, we decided nonetheless to perform independent T-tests [11]. A p-value of <0.05 is considered significant. The within participants comparison is analysed with a paired *T*-test.

## Results

The total average score of all the separate stations on the OSCE in 2011–2012 and 2012–2013 for year 1 and 2 combined was 3.58 ± 0.67 (n = 2010 individual student scores on station performance). The scores ranged from 2.00 to 5.00. The average score for all stations in 2011–2012 is 3.51 ± 0.65 (*n* = 954) and in 2012–2013 3.62 ± 0.69 (*n* = 1056) (t = −3.68, p < 0,01; Cohen’s d = 0.16). The average was significantly higher in the academic year in which more e-modules were available to prepare for training sessions. In our subsequent analyses we tried to unravel whether this difference could be attributed to the introduction of blended learning.

### Comparison of scores for skill stations with e-module preparation and stations with text based preparation (within cohort, different stations)

Students from the 2012–2013 cohort obtained an average score of 3.58 ± 0.67 (*n* = 554) on stations with text based preparation versus 3.67 ± 0.71 (*n* = 502) on stations with e-module preparation. The average scores of stations with an e-module preparation were significantly higher than the average scores of stations with text based preparation (t = 2.14 p = 0.03; Cohen’s d = 0.13) (see Table 2).

### Comparison of scores before and after introduction of mandatory e-module preparation (between cohorts, same station)

The average score on stations at the 2012 (cohort 2011–2012) and 2013 (cohort 2012–2013) examination showed that at the 2013 examination, students obtained a higher score on the stations which had moved from text based preparation in 2012 to e-module preparation in 2013; 3.44 ± 0.63 (*n* = 308) and 3.68 ± 0.70 (*n* = 314) (t = 4.97 p < 0.01; Cohen’s d = 0.36) respectively. This difference was significant (see Table 3).

The results showed no significant difference between the two cohorts on stations for which students had prepared by text based preparation in both academic years, 3.57 ± 0.67 (cohort 2011–2012, n = 467) and 3.61 ± 0.67 (cohort 2012–2013, n = 517), (t = 1.47 p = 0.34) . Thus, the higher score on the stations with e-module preparation was not a result of the cohort as a whole performing better on all stations.

### Within participants comparison (within student, different stations)

For 332 students we had two station scores with different preparation: one station with text based preparation and one with e-module preparation. The within-subject comparison showed a station average of 3.50 ± 0.68 for the stations with a text based preparation and an average of 3.60 ± 0.66 for stations with an e-module preparation. This difference was significant, (t = 2.48 p = 0.01; Cohen’s d = 0.15).

## Discussion

Our results showed that students obtained on average a higher score for stations with an e-module preparation before the training session than on stations with text based preparation before the training session. We have confidence in our results, because we performed several comparisons that support this conclusion. To begin with, we demonstrated that students at the 2013 OSCE, when more e-modules were available to prepare for the skills training sessions, scored on average higher than at the previous year’s OSCE, when fewer e-modules were available. Though this could theoretically be caused by the 2012–2013 cohort being better in general than its predecessor, within-cohort comparisons also revealed better scores on OSCE stations with e-module preparation than those without. As this comparison included different OSCE stations (different skills), we needed to corroborate these results by investigating OSCE scores of the same skills that moved from text based preparation to e-module preparation between 2012 and 2013 (between cohorts comparison). Again, we found better scores for skills with e-modules than for skills with written assignments. Finally, we compared within participant differences for those students who were tested on one skill with text based preparation versus a second skill with e-modules. Though this comparison involved different skills, the results were in line with the other findings: students showed better performance on skills for which they had prepared by e-modules. Our findings add to an earlier evaluation of mandatory e-modules as preparation for training sessions, which reports high satisfaction in both students and teachers [12]. In addition to this higher satisfaction, the current study found more objective evidence of e-module preparation actually being more effective in improving learning outcomes from skill training sessions.

The 2-station OSCE is a high-stakes examination for which students will prepare extensively. Students must pass this examination before they are allowed to start with their clinical rotations. In this context, it would not be very realistic to expect large effects of the substitution of text based preparation by mandatory e-modules, which is a relatively small intervention given that students have many more options to practice the skills. Yet, we were still able to demonstrate a significant effect. And although the average improvement may be quantitatively small, ranging from 0.09 (comparison 1) to 0.24 (comparison 2) on a 5-point scale, a difference of this size for the whole cohort may have a relevant impact on numbers of students failing the exam. It might be expected that in a large number of students many students score around the pass/fail cutoff. Therefore, a small improvement can result in an increase in the number of students passing the exam.

It might be argued that our results were an artifact of e-modules being developed for relatively easy skills, on which students already showed better performance across the board. However, our results suggested the opposite, i.e. e-modules were developed for the more difficult stations. In 2011–2012 students obtained lower scores on the OSCE stations for which e-module preparation was developed in 2012–2013 than on the stations for which no e-modules were yet developed in 2012–2013 (Table 3). This was an accidental finding, because there was no faculty policy to prepare e-modules for more difficult skills first, but it substantiated our results. This suggests that our study may underestimate the actual improvement caused by the introduction of e-module preparation.

Our study has a few limitations, though. First, the ideal study design to test an effect of this form of blended learning would be a controlled trial. However, for practical reasons such a design would not be possible as it would imply randomly assigning half of the students to an e-module preparation condition, and the other half to a text based condition. Such randomization should be made per training group to optimize expected gain in training time, but in practice students frequently change groups. Besides, students exchange information and it would be impossible to prevent students from using e-modules anyway. In an effort to overcome this limitation, we performed the abovementioned checks on the results. Because the study was not a controlled trial, the OSCE examiners were not blinded with respect to the availability of e-modules for the skills they assessed. Theoretically, whether or not students had e-module preparation could have influenced the scores the examiners assigned. Four of the seven examiners were also the developers of the e-modules. However, this would be far-fetched, if only because at the time of the examination the examiners were unaware that the results would be used to assess the effectiveness of the preparation for skill training sessions. We have no reason to expect any relationship between e-module preparation and students’ assignment to OSCE stations.

Second, the within student comparison showed better performance on stations which had e-module preparation versus text based preparation. However, it should be noted that the 2 OSCE stations were included in one OSCE time slot. Hence, one skill was always tested first, followed by the second skill. This implied that if a student took much time to perform the first skill, less time would be available to perform the second skill. We found after further analysis of our data that in this comparison, skills with e-module preparation were more often tested first than skills with text based preparation and hence, these latter skills had a somewhat higher probability of not being completed by a student because of lack of time, which might result in poorer performance. In this within participant comparison, our results indeed showed lower performance on skills tested as second station, which can, at least partly, be attributed to lack of time to complete the second station. This effect only occurred in the within students comparison; the other comparisons were not affected by this potential bias, because first and second tested skills had equally often e-module and text based preparation.

Third, our intervention can be conceived as the introduction of a form of blended learning, and we cannot distinguish between the effects of three different aspects of the intervention: the fact that the E-module preparation has been made “obligatory” (i.e. unlike the text based preparation, we checked whether students went through the module), the richer format and the organization of the e-module (which was quite different from the text based preparation), and the fact that trainers changed their approach during the actual training sessions when the students had prepared by e-modules. For example, trainers had a strong impression they had to spend less time explaining the skill to the students and as a result, students had more time available to practice the skills. It is very likely that all these elements, mentioned above, reinforced each other and together contributed positively to the results. That is, we saw a spectacular increase in the number of students who indicated in the student evaluations that they prepared well (from 72 % not preparing to 2 % not preparing), and also the time they spent for the preparation increased substantially (from an average of 40 min per student in case of text based preparation to 69 min when they used e-modules). In addition, students pointed out receiving immediate feedback on their answers as very positive aspect of the e-modules. They missed this in the written assignments of the text based preparation. This could have improved their motivation to prepare more thoroughly. Teacher evaluations indicate that the training sessions became more effective, since the students were better prepared and more motivated, because they were already familiar with the physical examination skills, at least at a theoretical level. Thus, it remains an open question what caused the improvement, whether it was the content of the e-modules or some collateral factor, such as motivation or time on task.

Skills education has an important place in medical education. With the growing attention to the importance of patient safety and qualified personnel in healthcare, its role is expected to increase even further. Extensive practical skills training is often limited due to resources; this type of education has a high teacher-student ratio and is resource intensive. Increasing the effectiveness of skills training without prolonging costly training time, can therefore be very valuable for medical education. Our results show obligatory e-modules before practical skills training sessions improves learning of physical examination skills in first and second year medical students, and hence contribute to better preparation of students for their first clerkships. Based on these results it is certainly worthwhile for medical faculties to consider introducing a blended learning approach for practical skills training to enhance efficient use of this time and labour intensive type of education.

Further research is needed to answer the question whether the improved performance is due to the content of the e-modules or to their obligatory character and to find the most effective and efficient way to teach practical medical skills.

## Conclusion

In conclusion, our results show that results of skills training can be improved, by the introduction of e-modules without increasing teacher time.

## Appendix

Overview of skill training sessions in the medical curriculum UMC Utrecht Faculty of Medicine in year 1 and 2: 16 physical examination training sessions and 11 other basic medical skills training sessions (see Table 4).

**Table 4:** Skill training sessions year 1 and year 2

	Skill training session	PE/BMS		Skill training session	PE/ BMS
	Year 1			Year 2	
1.1	General examination and measuring blood pressure	PE	2.1	Repetition of neurological examination	PE
1.2	First aid	BMS	2.2	Joint examination: Knee and ankle	PE
1.3	Examination of head and neck	PE	2.3	Joint examination: Wrist and hand, repetition of elbow	PE
1.4	Joint examination: Elbow and shoulder	PE	2.4	Giving injections intramuscular and subcutaneous	BMS
1.5	Neurological examination: Inspection, tonus, strength	PE	2.5	Gynecology	BMS
1.6	Neurological examination: Reflexes, sensibility	PE	2.6	Resuscitation 2	BMS
1.7	Examination of the heart	PE	2.7	Repetition of abdominal examination	PE
1.8	Examination of the lungs	PE	2.8	Intravenous line	BMS
1.9	Abdominal examination: inspection, auscultation and percussion	PE	2.9	Examination of ear	BMS
1.10	Abdominal examination: percussion and palpation	PE	2.10	Repetition of examination of heart and lungs	PE
1.11	Cleaning and stitching wounds	BMS	2.11	Eye examination	BMS
1.12	Joint examination: Hip and spine	PE	2.12	Obstetrics	BMS
1.13	Venipuncture (2 training sessions)	BMS			
1.14	Resuscitation and AED	BMS			
1.15	Examination of blood vessels	PE			

*PE* physical examination

*BMS* basic medical skill
